# Sex-biased associations between gut microbiota and hematologic toxicity of neoadjuvant chemoradiotherapy in locally advanced rectal cancer

**DOI:** 10.1097/JS9.0000000000004688

**Published:** 2026-01-13

**Authors:** Xin Sui, Chen Shi, Maxiaowei Song, Wenyi Jiang, Shuai Li, Jianhao Geng, Jun Xu, Yangzi Zhang, Xianggao Zhu, Yong Cai, Bo Li, Hongzhi Wang, Dezuo Dong, Huajing Teng, Yongheng Li, Weihu Wang

**Affiliations:** a Key Laboratory of Carcinogenesis and Translational Research (Ministry of Education/Beijing), Department of Radiation Oncology, Peking University Cancer Hospital & Institute, Beijing, China; b Department of Cell Biology, School of Basic Medical Sciences, Peking University Stem Cell Research Center, Peking University Health Science Center, Peking University, Beijing, China; c State Key Laboratory of Holistic Integrative Management of Gastrointestinal Cancers, Beijing Key Laboratory of Carcinogenesis and Translational Research, Department of Radiation Oncology, Peking University Cancer Hospital & Institute, Beijing, China

**Keywords:** gut microbiota, hematologic toxicity, neoadjuvant chemoradiotherapy, rectal cancer, sex-biased difference

## Abstract

**Background:**

Compared to male cancer patients, female patients have a higher incidence and severity of adverse events (AEs) associated with anticancer treatment. The mechanism underlying these disparities is largely unknown, especially in the context of radiotherapy. Notably, the composition and metabolism of the gut microbiota differ between sexes, and such differences have been implicated in multiple physiological and pathological processes. In this study, we aimed to investigate the sex differences in hematologic toxicities during chemoradiotherapy in patients with locally advanced rectal cancer (LARC) and to explore the potential role of the gut microbiota in mediating these differences.

**Materials and Methods:**

This real-world study included 329 patients with LARC receiving neoadjuvant chemoradiotherapy (nCRT). Hematologic AEs were evaluated according to Common Terminology Criteria for Adverse Events 5.0. 16S rRNA sequencing, metatranscriptome sequencing, and metabolome detection were performed on longitudinal fecal samples. Mendelian randomization analyses were performed to investigate the correlation between stem cell and gut microbial traits.

**Results:**

In this cohort, females had a higher severity of hematologic AEs than males. Using integrated longitudinal multi-omics data, we identified sex-biased bacterial species (e.g., *Parasutterella excrementihominis* and *Bifidobacterium adolescentis*) and microbiota-mediated arginine metabolism associated with hematologic toxicities. Gut microbiome-mediated arginine metabolism is associated with an abundance of hematopoietic stem cells and may be involved in the occurrence of sex-biased radiotherapy-induced hematologic AEs.

**Conclusion:**

These results indicate that the differences in gut microbial composition and metabolism between the two sexes are associated with the sex-biased hematologic AEs induced by nCRT. Females require greater attention regarding the hematologic toxicity of chemoradiotherapy, and that gut microbes may serve as potential targets for sex-tailored strategies to restore hematopoietic function following chemoradiotherapy.

## Introduction

Growing evidence shows that female patients have a higher incidence and severity of adverse events (AEs) associated with cancer treatment, including chemotherapy, targeted therapy, and immunotherapy, than their male counterparts^[1–4]^. These sex-biased differences underscore the need for personalized therapy that considers sex as a key variable to optimize outcomes and reduce toxicity. In the radiotherapy group, sex disparities in AEs were observed. In the CAO/ARO/AIO-94 phase 3 trial, in which patients with locally advanced rectal cancer (LARC) received pre- or postoperative chemoradiotherapy, women had a higher risk of developing leukopenia, anemia, and intestinal injury^[5,6]^. A retrospective study on LARC patients treated with neoadjuvant chemoradiotherapy (nCRT) also showed a higher frequency of grade 3 or higher acute toxicities in women, mainly perianal dermatitis and diarrhea^[7]^.

The underlying causes of these sex-biased differences are complex. This may be due to differences in reported AEs, total dose received, or adherence to therapy. Biologically, the differences in sex hormones, genetics, and epigenetics may have an impact on the dimorphism of treatment toxicities^[8]^. Pharmacokinetics and pharmacodynamics differ between sexes, and males have a relatively higher elimination capacity of anticancer drugs, such as paclitaxel and fluorouracil^[9–11]^. In terms of radiotherapy, by analyzing DNA damage markers and chromosomal aberrations of irradiated lymphocytes, Schuster *et al* showed that there is no intrinsic difference in radiosensitivity between men and women^[12]^. Other unknown factors contributing to the sex disparity in cancer treatment toxicities are to be investigated.

The gut microbiota has been reported to be involved in the efficacy and toxicity of cancer treatments^[13,14]^. Sex dimorphism in microbiome composition, which mediates sex differences in metabolism, has been described in both humans and mice^[15]^, and its contribution to host immunity is related to autoimmune diseases and antitumor immunity^[16–19]^. Recently, sex-biased gut microbiome and metabolites have been reported to aggravate cancer development^[20]^. However, the link between sex-biased gut microbes and sexual dimorphism in cancer therapy or treatment-related AEs remains largely unclear.

In this real-world study, we observed a higher risk of developing hematologic AEs in female patients with LARC who received nCRT than in male patients. Based on a longitudinal multi-omics analysis of 353 fecal samples, we found an association between sex-specific microbiome characteristics and sex-biased hematologic AEs, and we further highlighted the role of arginine metabolism in the development of sex-biased hematologic AEs. This cohort study has been reported in line with the STROCSS guidelines^[21]^.

## Materials and methods

### Ethics

This study was approved by the Ethics Committee of our hospital (2018YJZ40). Written informed consent was obtained from all patients.

Patients and sampling

This is a single-center cohort study. Patients diagnosed with locally advanced rectal adenocarcinoma between January 2018 and November 2019 were prospectively enrolled. Sex was defined as that assigned at birth. The inclusion criteria were as follows: (1) 18–80 years old; (2) pathologically diagnosed rectal adenocarcinoma, planned to receive nCRT; (3) no prior anticancer therapy; and (4) signed informed consent and willingness to comply with follow-up. Exclusion criteria were as follows: (1) history of malignancy at other sites and (2) presence of autoimmune diseases. This was a real-world, consecutively enrolled cohort study. All eligible patients were included in the study. Therefore, no formal sample size calculations were performed.

The patients were treated with nCRT. Intensity-modulated radiotherapy was delivered to the planning gross target volume at 50–50.6 Gy and to the planning target volume at 41.8–45 Gy in 22–25 fractions. Concurrent chemotherapy consisted of CapeOx (oxaliplatin 85 mg/m^2^ intravenously every 2 weeks plus capecitabine 825 mg/m^2^ orally twice daily, 5 days/week) or capecitabine alone. Patients undergoing consolidation chemotherapy received two to four cycles of CapeOx or capecitabine following nCRT. Surgery, wait-and-watch, or systemic treatment was administered according to the patient’s response to neoadjuvant treatment.

**HIGHLIGHTS**

- Female patients with locally advanced rectal cancer exhibited higher severity of neoadjuvant chemoradiotherapy-induced hematologic toxicity compared to males.
- Gut microbial composition and metabolic traits were correlated with hematologic adverse events (AEs).
- Sex-biased microbial species, functional profiles, and metabolites were associated with sex disparities in hematologic AEs.
- Microbes may be potential targets for sex-tailored prevention and alleviation of chemoradiotherapy-induced toxicities.

Routine blood examinations were performed weekly for each patient during treatment. For each individual patient, each grade (0, 1, 2, 3, or 4) of each type of hematologic AE, including leukopenia, lymphopenia, neutropenia, anemia, and thrombocytopenia, was assessed according to the Common Terminology Criteria for Adverse Events (CTCAE) Version 5.0. To comprehensively describe the overall severity of AEs in a given patient, the hematologic toxicity index (HTI) was calculated as $HTI=X1+\frac{X2}{1+X1}+\frac{X3}{\left(1+X1\right)\left(1+X2\right)}+\ldots +\frac{Xn}{\left(1+X1\right)\ldots \left(1+Xn-1\right)}$, based on the weighted sum of different types of AEs within a certain patient^[22]^. For example, a patient exhibiting five grade 2 toxicities will have a score of HTI = 2 + 2/(1 + 2) + 2/(1 + 2)(1 + 2) + 2/(1 + 2)(1 + 2)(1 + 2) + 2/(1 + 2)(1 + 2)(1 + 2)(1 + 2) ≈ 2.9877. This index integrates both the variety and severity of AEs in each patient, providing a single continuous measure of the overall toxicity burden, which allows comparisons of toxicity profiles across groups while accounting for multiple concurrent AEs. The collection, processing, and preservation of each fecal sample have been described in detail in our previously published article^[14]^.

### 16S rRNA sequencing and analysis

Genomic DNA from each fecal sample was extracted using a PowerSoil DNA Isolation Kit (MoBio Laboratories, Carlsbad, California, USA). The hypervariable V3–V4 region of the 16S rRNA gene was amplified using the primers 338F and 806R. Positive amplicons were paired-end sequenced (2 × 300 bp) using a MiSeq platform (Illumina, San Diego, California, USA). The V3–V4 region sequences for each sample were quality-filtered and analyzed using QIIME 2-2019.7^[23]^. The amplicon sequence variants (ASVs) were taxonomically classified against the Greengenes rRNA database^[24,25]^. Two alpha diversity indices (Shannon index and Inverse Simpson indices), Bray–Curtis dissimilarity, and principal coordinates analysis (PCoA) between samples were calculated using the R package *vegan* based on the normalized ASV table.

### Metatranscriptome sequencing analysis

Total RNA was extracted from each fecal sample using an OMEGA Soil RNA Kit (Omega Bioteck, Norcross, Georgia, USA). For metatranscriptome library construction, only samples with an RNA integrity number of ≥ 7.0 were included. rRNA was depleted from each sample using the Ribo-Zero Gold rRNA Removal Kit (Illumina, San Diego, California, USA), followed by library construction using the TruSeq RNA Library Prep Kit v2 (Illumina). Library insert length and integrity were assessed using Agilent 2100 prior to paired-end sequencing (2 × 150 bp) on an Illumina HiSeq platform.

Raw sequence reads were processed by filtering low-quality and human-contaminating reads using KneadDataV0.7.2, using the hg38 human genome as a reference. Reads were aligned and taxonomically assigned using the HUMAnN 2.0 pipeline^[26]^, and the abundance of microbial-derived pathways from MetaCyc, as well as Gene Ontology (GO) and Kyoto Encyclopedia of Genes and Genomes Orthology (KO) terms, were obtained for each fecal sample. LEfSe was used to identify taxonomic and functional changes associated with hematologic AEs in the gut microbiota^[27]^, while functional enrichment analysis was conducted to explore the potential biological roles of the gut microbiota linked to hematologic AEs. Co-occurrence network analysis was performed to investigate microbial interactions. NetShift analysis was used to identify potential microbial driver taxa associated with hematologic AEs^[28]^.

### Metabolites detection

Frozen fecal samples were thawed and mixed with 400 μl precooled methanol/acetonitrile (1:1, v/v). Following sonication at 4°C for 10 min, the mixture was incubated at −20°C for 60 min. The supernatant was collected after centrifugation at 13 000 rpm for 15 min, lyophilized, and stored at −80°C until redissolution in 150 μl of acetonitrile/water (1:1, v/v) solvent for liquid chromatography Q-exactive mass spectrometry (LC-QE-MS) analysis. Detailed procedures for LC-QE-MS detection were described in our previously published article^[14]^. Metabolite enrichment analysis was conducted with MBROLE3 (https://csbg.cnb.csic.es/mbrole3/index.php)^[29]^, using significantly altered metabolites identified from untargeted metabolomics data.

### Multivariable associations with linear model analysis

Multivariable associations with linear models (MaAsLin2) were used to identify associations among microbiome features, clinical variables, and hematologic AEs^[30]^. All input features, including species-level relative abundances, microbial metabolic pathways, and relative abundances of metabolites, were filtered and transformed with default parameters. Clinical variables, including specific hematologic AE, time point, age, sex, tumor location, tumor stage, and differentiation status, were regarded as fixed effects. Each detected association among microbiome features and specific hematologic AE or sex was adjusted for clinical variables, including time point, age, tumor location, tumor stage, and differentiation status. A limited number of repeated measurements per subject for species-level microbiome data in our cohort may lead to unstable estimation of the random-effect variance component. Thus, fixed effects were used in MaAsLin2 with default settings, and multiple testing was corrected using the Benjamini–Hochberg method to control the false discovery rate. To balance discovery and type I error control, according to the default setting of MaAsLin2, the q-value threshold for significance was set at 0.25^[30,31]^.

### Mendelian randomization analysis

Mendelian randomization (MR) analyses were performed using the R package *TwoSampleMR* to identify causal relationships between gut microbiota features and the activity of hematopoietic stem/progenitor cells. Summary-level data on the abundance of hematopoietic stem cells (HSCs) (ebi-a-GCST90001514) and hematopoietic progenitor cell antigen CD34 (prot-a-440) were downloaded from the IEU Open GWAS database using the R package *ieugwasr* as outcome data. These data were derived from two studies: one aimed at unveiling genetic signatures in immune cells that underlie autoimmunity^[32]^, and the other focused on the genomic atlas of the human plasma proteome^[33]^. In addition, we obtained summary-level data on the abundance of gut microbiota and arginine from the IEU Open GWAS database as exposure data. These data were derived from previous host genetics-based studies on the gut microbiome and blood metabolites^[34–36].^ The association inclusion threshold of the instrument variable was set at *P* < 1 × 10^−5^, and single-nucleotide polymorphisms (SNPs) were excluded if they were in linkage disequilibrium (LD) with one another (*r*^2^ > 0.01) within a 1000 kb window. In the absence of shared SNPs between exposure and outcome, LD proxies with a minimum *r*^2^ of 0.8 were utilized. Three methods, inverse variance-weighted, MR-Egger, and weighted median MR, were used for two-sample MR analyses. We additionally conducted multiple sensitivity analyses, including directional horizontal pleiotropy, heterogeneity, and leave-one-out analysis, to test the reliability of MR causal estimates.

Statistics

For the patients’ baseline characteristics, Fisher’s exact test was used for categorical variables. For normally distributed continuous variables, the Student’s *t*-test was applied, and for non-normally distributed continuous variables, the Mann–Whitney *U* test was used. A two-sided *P*-value of less than 0.05 was considered statistically significant. To compare alpha diversity indices between groups and across time points, statistical significance was determined using the Scheirer–Ray–Hare test.

## Results

### Increased severity of hematologic AEs in female than male patients with LARC following nCRT

Three hundred and twenty-nine patients with LARC who underwent nCRT at our department between January 2018 and November 2019 were enrolled in this study. The clinical baseline characteristics are presented in Table 1. Treatment-attributable objectively measured hematologic AEs, including leukopenia, lymphopenia, neutropenia, anemia, and thrombocytopenia, were assessed according to the CTCAE Version 5.0. Three hundred and twenty-three patients (98.18%) experienced at least one grade ≥ 1 hematologic AE. The most common AE was lymphopenia (97.27%), and 151 patients (45.90%) had grade 3 or 4 lymphopenia. Female patients had a significantly higher severity of leukopenia, lymphopenia, and neutropenia (Table 1). To comprehensively describe the overall severity of AEs in a given patient, HTI was calculated based on the weighted sum of different types of AEs within a certain patient^[22]^ and was used for further analysis. The severity of HTI was still higher in females than in males, confirming the higher severity of hematologic toxicity in female patients with LARC following nCRT (Table 1). Although the inclusion of oxaliplatin in the concurrent chemotherapy regimen enhanced the severity of lymphopenia, the use of oxaliplatin was balanced between male and female patients.

**Table 1:** Clinical characteristics potentially associated with hematological toxicity.

	Leukopenia			Lymphopenia			Neutropenia			Anemia			Thrombopenia			Hematologic Toxicity Index		
	0 *n* = 127	≥1 *n* = 202	*P* value	0–2 *n* = 179	3–4 *n* = 150	*P* value	0 *n* = 245	≥1 *n* = 84	*P* value	0 *n* = 263	≥1 *n* = 66	*P* value	0 *n* = 297	≥1 *n* = 32	*P* value	≤Median *n* = 164	>Median *n* = 165	*P* value
Age (years)	61.2	55.3	**0.000**	59.0	55.9	**0.007**	58.6	54.6	**0.002**	57.1	59.5	0.092	57.6	57.7	0.967	59.2	56	0.337
Sex			**0.000**			**0.009**			**0.002**			0.937			0.236			**0.002**
Male	106	132		140	98		188	50		190	48		212	26		131	107	
Female	21	70		39	52		57	34		73	18		85	6		33	58	
Tumor location			0.159			0.306			0.891			**0.047**			**0.009**			0.544
Low	81	113		101	93		145	49		148	46		182	12		94	100	
Mid-high	46	89		78	57		100	35		115	20		115	20		70	65	
Clinical T stage			0.846			0.406			0.870			**0.006**			0.832			0.265
cT1	0	1		1	0		1	0		1	0		1	0		1	0	
cT2	13	22		23	12		26	9		24	11		31	4		22	13	
cT3	87	140		120	107		167	60		193	34		207	20		111	116	
cT4	27	39		35	31		51	15		45	21		58	8		30	36	
Clinical N stage			0.14			**0.032**			0.104			0.619			0.736			**0.009**
cN−	14	13		20	7		24	3		23	4		24	3		20	7	
cN+	113	189		159	143		211	81		240	62		273	29		144	158	
Concurrent with oxaliplatin			0.854			**0.000**			0.246			0.834			0.066			**0.000**
−oxaliplatin	83	130		135	78		163	50		171	42		197	16		127	86	
+oxaliplatin	44	72		44	72		82	34		92	24		100	16		37	79	

*P* values less than 0.05 are shown in bold.

### Altered fecal microbial community associated with hematologic toxicity of LARC patients following nCRT

**Figure 1.**
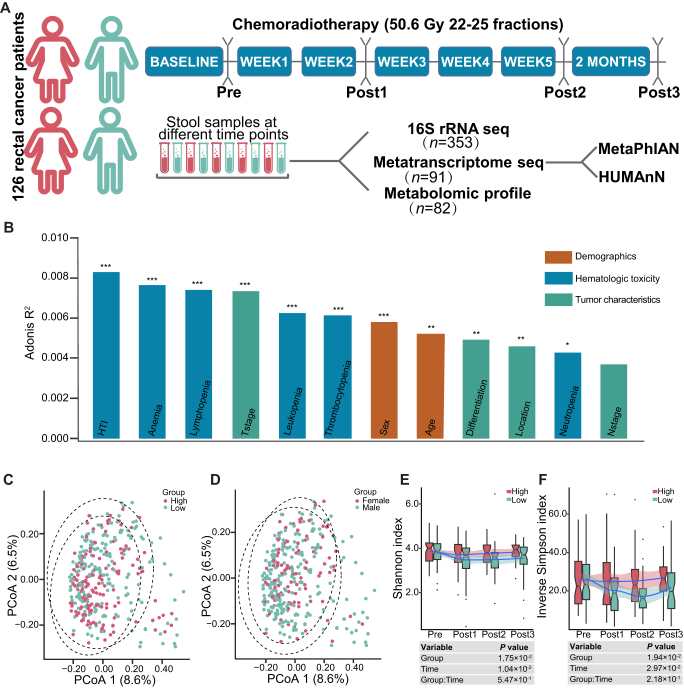
Experimental design and fecal microbial community associated with hematologic toxicity in patients undergoing neoadjuvant chemoradiotherapy. (A) Experimental workflow of this study. (B) Vertical bars showing the effect size (*R*^2^) of each variable on the composition of gut microbiome as determined via PERMANOVA analysis. ***P < 0.001; **P < 0.01; *P < 0.05. HTI: weighted hematologic toxicities. PCoA of fecal samples from locally advanced rectal cancer (LARC) patients with different severity of HTI (C) or sex (D) using Bray–Curtis distance at the amplicon sequence variant (ASV) level. Box-and-whisker plots display Shannon indices (E) and inverse Simpson indices (F) obtained by ASV of 16S rRNA in the fecal samples of LARC patients at four time points before and after nCRT. Statistical significance was calculated with the Scheirer–Ray–Hare test.

A total of 353 fecal samples were collected from a subset (*n* = 126) of patients with LARC at four time points before and after nCRT (Fig. 1A). The baseline characteristics of this subset and all 329 patients were comparable in most characteristics, except for the concurrent chemotherapy regimen (Supplemental Digital Content Table S1, available at: http://links.lww.com/JS9/G618). The percentage of oxaliplatin-based regimen was higher in the subset, and there was no significant difference in the distribution of concurrent chemotherapy regimen between the two sexes in this subset (*P* = 0.204) and among all patients (*P* = 0.591). Multi-omics analyses based on 16S rRNA sequencing, metatranscriptome sequencing, and metabolome profiling were performed^[14]^. Several variables, including clinical baseline characteristics and AEs that may affect the composition of the gut microbiome, were analyzed by permutational multivariate ANOVA analysis. Weighted hematologic toxicities were the strongest contributors to the compositional characteristics of the gut microbiome, while among the demographic parameters, sex explained the greatest proportion of microbial variance (Fig. 1B). PCoA showed different microbiome profiles between fecal samples with different severities of hematologic AEs (Fig. 1C) and between sexes (Fig. 1D). In patients with low HTI, microbial alpha diversity declined upon nCRT and showed a slow recovery after the completion of radiotherapy, whereas in patients with high HTI, the diversity remained stable during the neoadjuvant treatment process (Fig. 1E and F).

### Fecal microbes and metabolites correlated with hematologic AEs of LARC patients following nCRT

**Figure 2.**
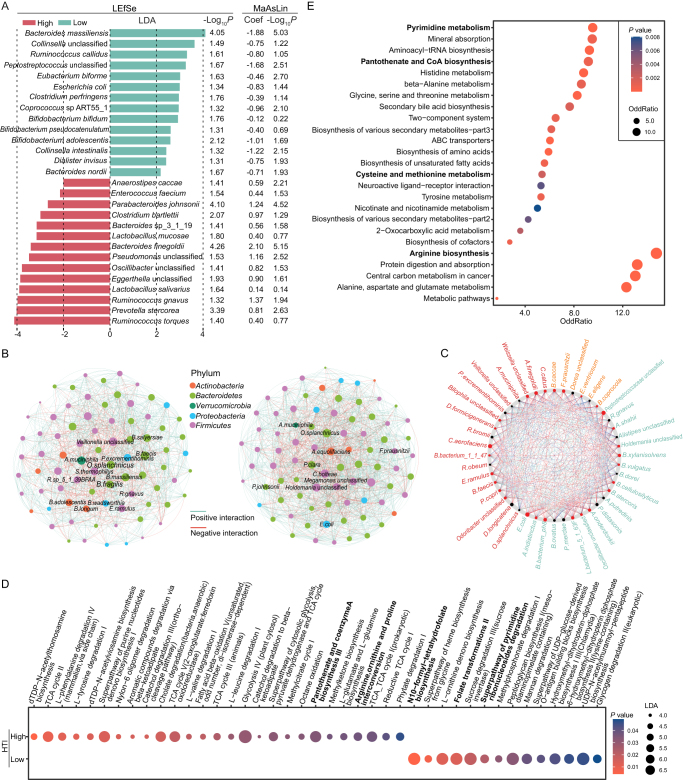
Correlation of fecal microbes or metabolites with hematologic AEs of LARC patients following nCRT. (A) Species significantly enriched in the high and low hematologic AE groups identified by LEfSe. The coefficients (Coef) and −Log_10_*P* values of these species, derived from multivariable association analysis with linear models (MaAsLin), are listed on the right. (B) Co-occurrence networks of the microbiome constructed based on post-treatment fecal samples of low (left) and high (right) hematologic toxicity groups. Nodes represent species, with their colors indicating their respective phyla, and node size reflecting the number of connections to other nodes. Edges colored green or red denote positive or negative microbial correlations, respectively. (C) Potential species driving the alteration of the microbiome structure from the low to high hematologic toxicity group. Node sizes are proportional to their scaled neighbor shift score^[28]^, and nodes colored red indicate an increase in their betweenness from the low to high hematologic toxicity group. Functional enrichment of transcriptome (D) and differentially abundant metabolites (E) in the feces of patients with high and low hematologic toxicity.

To systemically describe the associations between fecal microbes and their functions with hematologic AEs of nCRT, we analyzed the metatranscriptome and metabolome of the fecal samples. Feces from patients with high and low HTI were characterized by different microbial compositions (Fig. 2A). Feces in the low HTI group were enriched with probiotics, such as *Bifidobacterium adolescentis*, while those in the high HTI group were characterized by *Ruminococcus gnavus* and *Anaerostipes caccae*. As microbes function as an interactive network, we performed co-occurrence analysis to explore the fecal microbial community in patients with high and low HTI (Fig. 2B). The core microbes in the low HTI group were *Akkermansia muciniphila, Bacteroides faecis, Odoribacter splanchnicus, B. adolescentis*, etc.; while in the high HTI group, the core microbes were *Clostridium bolteae, Adlercreutzia equolifaciens*, and *Paraprevotella clara*, etc. NetShift analysis identified several bacteria, including *Collinsella aerofaciens, Alistipes finegoldii*, and *Faecalibacterium prausnitzii*, as potential driver taxa of the microbiome structure alteration between groups (Fig. 2C). The MetaCyc-annotated functional modules differed between the two groups. The high HTI group was enriched in the TCA cycle, purine nucleotides *de novo* biosynthesis, pantothenate and CoA biosynthesis, arginine, ornithine, and proline interconversion pathways. The low HTI group was enriched in N10-formyl-tetrahydrofolate biosynthesis, folate transformation, and pyrimidine ribonucleoside degradation pathways (Fig. 2D). In accordance with metatranscriptome analysis, metabolome analysis showed that arginine biosynthesis, pyrimidine metabolism, pantothenate and CoA biosynthesis, and cysteine and methionine biosynthesis were related to hematologic AEs severity (Fig. 2E). The transcription and metabolism profiles between the two groups highlight the role of arginine- and pantothenate-related functions in hematologic toxicity.

### Bacterial species linked to sex disparity in each type of therapeutic toxicities induced by nCRT

**Figure 3.**
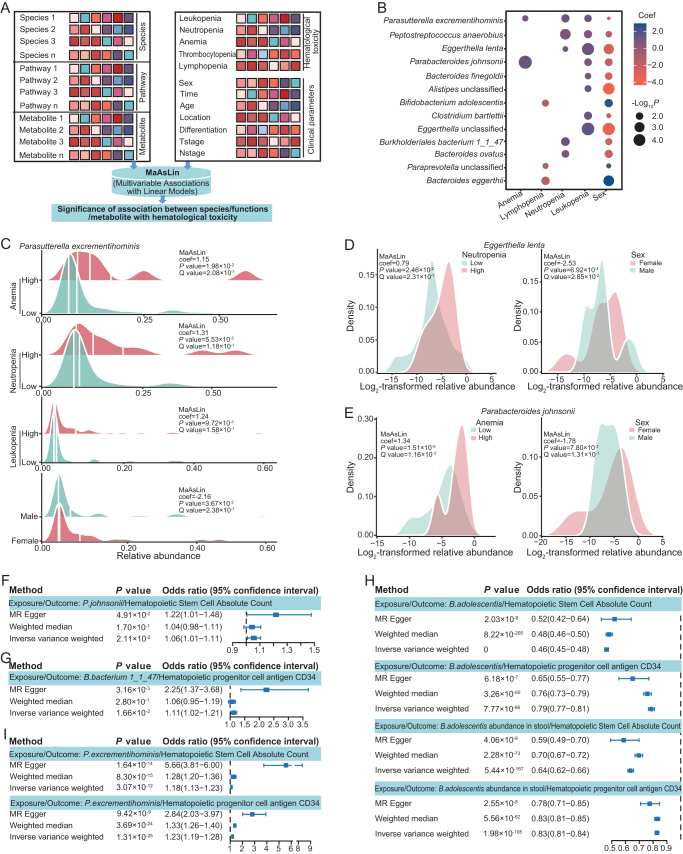
Bacterial species linked to sex disparity in each type of therapeutic toxicities induced by nCRT. (A) MaAsLin (Multivariable Associations with Linear Models) analysis was used to assess associations between microbial species, pathways, metabolites, and clinical parameters with hematologic AEs. (B) The dot plot shows the coefficients (coef) of gut microbial species with different hematologic AEs and sex. Relative abundance of *P. excrementihominis* (C), *E. lenta* (D) or *P. johnsonii* (E) in patients with indicated hematologic toxicities, and in males versus females. (F–I) Mendelian randomization (MR) analysis on the causal effect of indicated microbial species on counts of hematopoietic stem cells or CD34 on hematopoietic progenitor cells, based on a Dutch cohort^[34]^ and an Italian cohort^[32]^. The odds ratio and 95% confidence interval were obtained using the MR-Egger, weighted median, and inverse variance weighted methods.

As both the gut microbiome and sex were correlated with the severity of hematologic AEs, we explored the sex-associated gut microbiome characteristics that might contribute to the severity of each type of hematologic AEs. MaAsLin analysis was applied to comprehensively relate the microbial species, pathways, metabolites, and clinical parameters to hematologic AEs (Fig. 3A). Sex-specific gut microorganisms were positively or negatively correlated with the severity of hematologic AEs (Fig. 3B; Supplemental Digital Content Table S2, available at: http://links.lww.com/JS9/G618). *Parasutterella excrementihominis*, a species belonging to the *Parasutterella* genus that has been documented to be abundant in patients with Crohn’s disease and scarce in pediatric inflammatory bowel disease patients who show a good response to treatment^[37,38]^, was enriched in the female gut and was associated with a higher risk of anemia, leukopenia, and neutropenia (Fig. 3C). *Eggerthella lenta*, an anaerobic opportunistic pathogen enriched in patients with inflammatory bowel disease, can induce intestinal Th17 activation and worsen colitis in a *Rorc*-dependent manner in mice^[39,40]^, was also enriched in the female gut and was associated with a higher risk of neutropenia (Fig. 3D). As previously reported, the abundance of *Parabacteroides johnsonii* was higher in women than in men, and higher in pre-menopausal women than in post-menopausal women^[41]^. In our cohort, *P. johnsonii* also showed higher abundance in women and was significantly associated with a higher risk of nCRT-induced anemia (Fig. 3E).

The occurrence of hematologic AEs was mainly due to the repression of HSCs in the bone marrow^[42].^ We analyzed the potential causal relationship between the gut microbiome and the abundance of HSC utilizing the genetic version of the randomized controlled trials, MR, based on the collected cohort of host genetics on the gut microbiome and immune cells^[32,34]^. The abundance of three bacterial species identified in our LARC cohort, *P. johnsonii, Burkholderiales bacterium 1*_*1_47*, and *P. excrementihominis*, was negatively related to counts of HSC or CD34-positive hematopoietic progenitor cells (Fig. 3F, G, and I). In contrast, gut *B. adolescentis* showed a positive correlation with the counts of HSC or CD34 in hematopoietic progenitor cells (Fig. 3H). Notably, *B. adolescentis* was enriched in the male gut microbiome (Fig. 3B). It is also recognized as a probiotic and exerts competitive metabolic interactions with *E. lenta*^[43]^, a female gut-enriched species identified in our study (Fig. 3D). Based on these results, we inferred that *B. adolescentis* might play a role in mitigating nCRT-induced hematologic AEs in males, although further experimental evidence is needed in future studies.

### Sex-biased functional profiles and metabolites associated with therapeutic toxicities in LARC patients following nCRT

**Figure 4.**
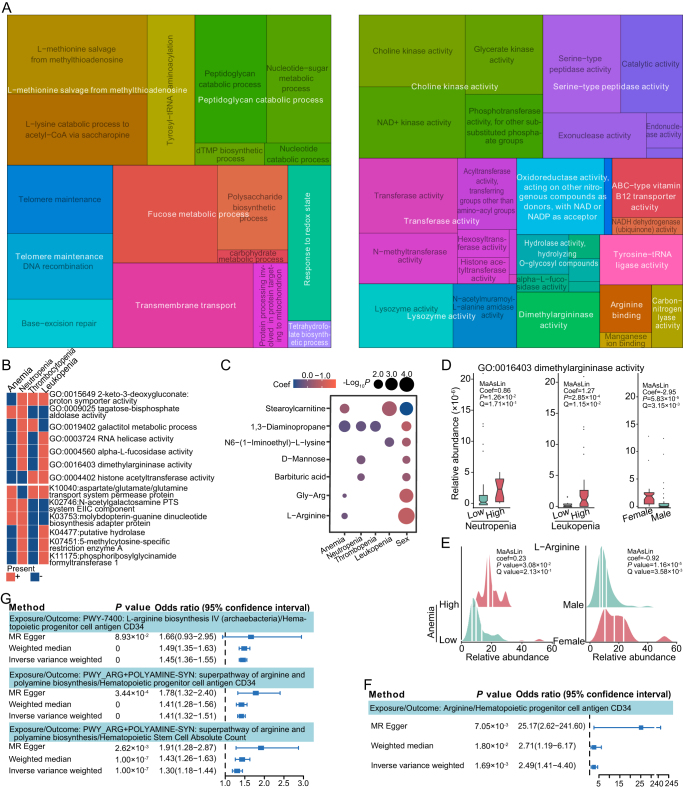
Sex-biased functional profiles and metabolites associated with therapeutic toxicities in LARC patients following nCRT. Sex-biased Gene Ontology (GO) terms associated with each specific hematologic AEs were summarized and visualized based on semantic similarity using the R package *rrvgo*. Left, biological process domain; right, molecular functions domain. (B) Functional enrichment differences annotated by GO or KEGG in different types of hematologic AEs. (C) The dot plot shows the coefficients (coef) of sex-associated metabolites with different hematologic AEs. The abundance of dimethylargininase activity (D) and l-arginine (E) between groups stratified by different hematologic AEs or sexes. Mendelian randomization (MR) analysis on the causal effect of the arginine (F) and arginine-related metabolic pathways derived from the gut microbiota (G) on counts of hematopoietic stem cells or CD34 on hematopoietic progenitor cells. The Odds ratio and 95% confidence interval were obtained using the MR-Egger, weighted median, and inverse variance weighted methods.

Next, we investigated the metabolic pathways associated with sex-biased hematologic AEs based on MaAsLin analysis. Sex-biased GO terms associated with each specific hematologic AEs were summarized and visualized based on semantic similarity (Fig. 4A). In the biological process domain, the roles of l-methionine salvage from methylthioadenosine, peptidoglycan catabolic process, telomere maintenance, fucose metabolic process, transmembrane transport, response to redox state, and tetrahydrofolate biosynthetic process were highlighted. In the molecular function domain, choline kinase, transferase, lysozyme, serine-type peptidase, oxidoreductase, dimethylargininase, and arginine binding are involved in the development of sex-biased hematologic AEs. Regarding specific hematologic AEs, functional enrichment annotated by GO or Kyoto Encyclopenia of Genes and Genomes (KEGG) showed differences between the different types of AEs (Fig. 4B; Supplemental Digital Content Table S3, available at: http://links.lww.com/JS9/G618, Supplemental Digital Content Table S4, available at: http://links.lww.com/JS9/G618). Among these, neutropenia showed a distinct sex-associated pattern of microbial functional traits, featured by elevated activities of dimethylargininase, histone acetyltransferase, RNA helicase, and alpha-l-fucosidase. MaAsLin analysis identified metabolites related to sex-biased hematologic AEs. Levels of fecal l-arginine and Gly–Arg were higher in female patients and associated with a higher risk of anemia (Fig. 4C; Supplemental Digital Content Table S5, available at: http://links.lww.com/JS9/G618). dl-alpha-hydroxybutyric acid and Gly–Lys were also enriched in female patients and were associated with a higher HTI (Fig. 4C). Dimethylargininase metabolizes asymmetric dimethylarginine (ADMA), an endogenous arginine derivative, into citrulline and the corresponding alkylamine, and plays a crucial role in regulating nitric oxide levels^[44]^. In our cohort, gut microbial dimethylargininase activity was higher in women and was correlated with a higher percentage of neutropenia and leukopenia after nCRT (Fig. 4D). As a precursor of nitric oxide, l-arginine has been used as a coadjuvant in the treatment of sickle cell anemia for pain relief^[45,46]^. Our study showed that fecal l-arginine was relatively abundant in females and in patients who developed anemia upon nCRT (Fig. 4E). MR analysis showed a negative correlation between the count of HSC or CD34 on hematopoietic progenitor cells and arginine abundance (Fig. 4F), l-arginine biosynthesis, or superpathway of arginine and polyamine biosynthesis derived from the gut microbiota (Fig. 4G). Taken together, our data suggest that gut microbiome-mediated arginine metabolism may be involved in the occurrence of sex-biased nCRT-induced hematologic AEs.

## Discussion

Compared with males, females experience more AEs from cancer treatment, including chemotherapy, targeted therapy, immunotherapy, and radiotherapy^[1-7]^. Females have a higher incidence of radiotherapy discontinuation or interruption^[47,48],^ which may compromise tumor control. In this real-world study of 329 LARC patients receiving nCRT, we found that female patients had a higher risk of developing hematologic toxicity, highlighting the need for increased clinical awareness of female patients in practice.

Hematopoietic cells are sensitive to radiation and chemotherapy, and hematologic AEs mainly stem from the suppression of HSCs in the bone marrow^[42]^. Gut microbiota are involved in the process of hematopoiesis^[49]^, and germ-free or antibiotics-treated mice show defects in bone marrow function^[50]^. Studies on mice show that the gut microbiota holds the potential for radioprotection. The fecal engraftment and dirty cage sharing from “elite-survivors” could provide radioprotection to germ-free and conventionally housed mice, and the protective role of *Lachnospiraceae*, short-chain fatty acid and tryptophan metabolites was identified. Treatment with bacteria or metabolites ameliorated radiation-induced toxicity in mice by restoring bone marrow cellularity and gut integrity^[51]^. In patients with colorectal cancer, the composition of gut microbiota differs between sexes^[52]^. Thus, we explored the composition and metabolism of gut microbiota, which may serve as a bridge between sex dimorphism and hematologic AE to understand the sex differences in nCRT-related hematologic toxicity.

In the present study, for patients with low HTI, the gut microbiota was enriched in probiotics, such as *B. bifidum* and *B. adolescentis* (Fig. 2A). *B. bifidum* has been reported to restore hemoglobin levels in patients with renal disease undergoing hemodialysis^[53]^, and modulate the immune status by increasing the B cell population, maintaining CD4^+^ lymphocytes, and producing a lower inflammatory cytokine profile when administered to the elderly population^[54,55]^. Thus, beneficial microbes may be involved in restoring bone marrow function upon chemoradiotherapy. In contrast, some microbes enriched in the HTI group, such as *R. gnavus* and *Ruminococcus torques* (Fig. 2A), were primarily considered to be associated with the pathogenesis of inflammatory bowel disease by exerting a pro-inflammatory role^[56–58]^. Their role in the incidence of hematologic toxicity upon chemoradiotherapy needs to be determined in future studies. Moreover, using MaAsLin analysis, we identified microbes enriched in a specific sex that were associated with hematologic toxicity of nCRT (Fig. 3A**–**E). *P. excrementihominis, E. lenta*, and *P. johnsonii* were enriched in the female gut microbiota and positively correlated with the severity of hematologic toxicity, and the increased abundance of *P. excrementihominis* or *P. johnsonii* was inferred to be associated with the decreased abundance of HSCs or progenitor cells (Fig. 3F and G). In contrast, *B. adolescentis* was enriched in the male gut microbiome and was negatively correlated with the severity of hematologic toxicity (Fig. 3B). *B. adolescentis* has been reported to reduce inflammation^[59]^ and boost catalase activity to process the active oxygen metabolism, resulting in an improved healthspan and lifespan^[60]^. MR analysis revealed the protective role of *B. adolescentis* in restoring the count/activity of HSCs and progenitor cells (Fig. 3H). These results shed light on the underestimated role of sex-specific gut microbiota in chemoradiotherapy-induced hematological toxicity. *B. adolescentis* is recognized as a probiotic, and its administration was considered safe and has a positive effect on gut homeostasis maintenance and immune modulation^[61,62]^. Supplementation of *B. adolescentis* may potentially prevent or alleviate chemoradiotherapy-induced hematologic toxicities, especially in females, although more clinical evidence is still needed.

The gut microbiota exerts effects on host physiology, mainly through the production and modulation of bioactive metabolites. Among the metabolites related to hematologic toxicity, especially in females, the arginine pathway was stably detected in multi-omics assays (Fig. 4A–E). l-arginine can be converted into nitric oxide by nitric oxide synthase. A placebo-controlled trial showed that administering l-arginine before each fraction enhanced radiotherapy efficacy, and preclinical studies have demonstrated that l-arginine-induced radiosensitization stems from enhanced vulnerability to DNA damage in specific cancer cells undergoing nitric oxide-induced metabolic suppression^[63]^. ADMA is an endogenous nitric oxide synthase inhibitor, and its degradation by dimethylargininase restores the production of nitric oxide. The activity of dimethylargininase is associated with estrogen levels^[64]^, and it was enriched in female gut microbiota and positively correlated with the severity of neutropenia and leukopenia (Fig. 4D). Nitric oxide is a negative regulator of HSC activity, migration, and hematopoiesis^[65–68]^, and in turn, suppressing the activity of nitric oxide synthases may increase bone marrow stem and progenitor cell numbers^[67]^. In our study, MR-based causal inference revealed a negative correlation between the count of HSC or CD34 on hematopoietic progenitor cells and arginine abundance (Fig. 4F) or gut microbiota-derived l-arginine biosynthesis (Fig. 4G). Although further experimental evidence is required, and currently the evidence of how gut microbiota-derived arginine enters circulation is still lacking, our data suggest that gut microbiota-mediated arginine metabolism may contribute to sex-biased nCRT-induced hematologic AEs via the nitric oxide pathway. A study on a mouse model showed that an arginine-restricted diet had a different impact on female and male mice. Nitric oxide synthase activity was not altered in the arginine-restricted diet in male mice, but was significantly reduced in female mice^[69]^. Modulating arginine intake may be a potential way to manage the hematologic toxicity in cancer treatment, after its impact on normal physiological function being precisely examined on preclinical models and clinical trials.

This study had several limitations that should be acknowledged. First, our microbial profiling focused solely on bacterial communities without incorporating other important components of the human microbiome, such as fungi, archaea, and viruses. These microbial groups are increasingly recognized as integral parts of the host microbiota ecosystem, with emerging evidence suggesting that they can modulate immune function, inflammation, and even treatment response, sometimes in a sex-specific manner^[70–72]^. The exclusion of these microbial domains may limit the completeness of our interpretation, particularly in the context of sex-related microbial differences. We are currently exploring the role of these components in these samples and hoping to give a more comprehensive description of the gut microecosystem in the setting of LARC receiving nCRT. Second, the findings in this study were primarily based on observational sequencing data from clinical samples, and mechanistic inferences have not been experimentally validated, which limits the ability to establish causality. Future studies incorporating functional assays are essential to confirm the roles of candidate microbes and metabolites and to elucidate the underlying mechanisms. Third, the effect of host genetics and other potential confounders on the composition of gut microbiota, hematopoietic system and treatment-related hematologic toxicity, including dietary information, body composition fluctuation, and other lifestyle factors, was not addressed due to the lack of information. Detailed genetic information and a questionnaire should be included in our further investigation on the gut microbiome. Another point to be noted is that these observations were obtained in LARC patients receiving nCRT. Considering the specificity of gut microbial profile of LARC patients, and the effect of treatment on gut microbiome, this finding may not be directly generalized to other types of malignancy and treatment modalities. Nevertheless, our findings highlight the potential role of gut microbiome on sex-biased nCRT-induced hematologic toxicities, and may provide clues for the prevention of toxicities in other cancer treatment settings.

## Conclusion

In summary, in this real-world study, we found that female patients with LARC exhibited increased severity of nCRT-induced hematologic toxicity compared to males, and our longitudinal multi-omics data identified sex-biased bacterial species, microbiota-derived functional profiles, and metabolites linked to such toxicities in these patients. Our results suggest females require greater attention regarding hematologic toxicity following chemoradiotherapy, and microbes may act as potential targets for sex-tailored restoration of bone marrow function after chemoradiotherapy.

## Data Availability

16S rRNA amplicon and metatranscriptome sequencing data were deposited in the Genome Sequence Archive in the National Genomics Data Center, China National Center for Bioinformation/Beijing Institute of Genomics, Chinese Academy of Sciences. The website is https://ngdc.cncb.ac.cn/gsa-human, and the accession number is HRA001813. Any additional information required to reanalyze the data reported in this paper is available from the corresponding authors upon request.

## Research registration unique identifying number (UIN)

Research Registry (researchregistry11562).
